# Next generation proteomics with drug sensitivity screening identifies sub-clones informing therapeutic and drug development strategies for multiple myeloma patients

**DOI:** 10.1038/s41598-021-90149-y

**Published:** 2021-06-18

**Authors:** Ciara Tierney, Despina Bazou, Muntasir M. Majumder, Pekka Anttila, Raija Silvennoinen, Caroline A. Heckman, Paul Dowling, Peter O’Gorman

**Affiliations:** 1 Department of Biology, National University of Ireland, Maynooth, Ireland; 2 Department of Hematology, Mater Misericordiae University Hospital, Dublin, Ireland; 3 Institute for Molecular Medicine Finland FIMM, Helsinki Institute of Life Science, University of Helsinki, Helsinki, Finland; 4 Department of Hematology, Helsinki University Hospital and Comprehensive Cancer Center, Helsinki, Finland

**Keywords:** Proteomics, Myeloma, Translational research

## Abstract

With the introduction of novel therapeutic agents, survival in Multiple Myeloma (MM) has increased in recent years. However, drug-resistant clones inevitably arise and lead to disease progression and death. The current International Myeloma Working Group response criteria are broad and make it difficult to clearly designate resistant and responsive patients thereby hampering proteo-genomic analysis for informative biomarkers for sensitivity. In this proof-of-concept study we addressed these challenges by combining an ex-vivo drug sensitivity testing platform with state-of-the-art proteomics analysis. 35 CD138-purified MM samples were taken from patients with newly diagnosed or relapsed MM and exposed to therapeutic agents from five therapeutic drug classes including Bortezomib, Quizinostat, Lenalidomide, Navitoclax and PF-04691502. Comparative proteomic analysis using liquid chromatography-mass spectrometry objectively determined the most and least sensitive patient groups. Using this approach several proteins of biological significance were identified in each drug class. In three of the five classes focal adhesion-related proteins predicted low sensitivity, suggesting that targeting this pathway could modulate cell adhesion mediated drug resistance. Using Receiver Operating Characteristic curve analysis, strong predictive power for the specificity and sensitivity of these potential biomarkers was identified. This approach has the potential to yield predictive theranostic protein panels that can inform therapeutic decision making.

## Introduction

Multiple myeloma (MM), the second most prevalent haematological malignancy^1^, has seen dramatic improvements in therapeutic options over recent years^2^. In spite of the rapid development of new drugs, evolution of multi-drug resistant clones remains inevitable^3^. One of the challenges of drug development is identifying the protein phenotype of resistant clones. Advances in proteomic technology offer the opportunity to identify effector proteins that directly modulate biological processes that lead to the emergence of resistant sub-clones. The study of malignant plasma cell samples from MM patients presents several challenges in terms of defining sensitive versus resistant cohorts. For example, the current international myeloma working group (IMWG) criteria for assessing response to treatment are broad with overlap between different groups^4^. Without clear objective delineation between sensitive and resistance groups comparative proteomic statistical analysis is weakened making it difficult to clearly identify a resistant protein phenotype. Similarly, the use of triplet combinations as standard of care makes it difficult to identify resistance to individual drugs^5^.

Personalised medicine is predicted to be the future of treatment strategies for MM patients. Precise malignant plasma cell phenotyping and genotyping using a combination of Omics techniques offers the tantalising prospect of an individualised theranostic profile that provides a sensitivity profile for each drug class^6^.

The proteasome inhibitor (PI), Bortezomib, a first in class, reversible boronic acid dipeptide with high selectivity for inhibition of the 26S proteasome, induces mitochondrial depolarisation and apoptosis. Bortezomib binds to the catalytic site of the 26S proteasome resulting in an abundance of p53 and p27 and an inhibition of Nuclear Factor kappa B (NFkB) transcriptional activity^7^, leading to increased cell stress and apoptosis^8^.

The immunomodulatory drug (IMiD) Lenalidomide increases T cell proliferation^9^ and by inhibiting tumour necrosis factor-alpha (TNFα)-induced endothelial cell migration, basic Fibroblast Growth Factor (bFGF) and Vascular Endothelial Growth Factor (VEGF), it exhibits anti-angiogenic properties^10^.

The B-cell lymphoma 2 (BCL/2) inhibitor, Navitoclax, is a high affinity small molecule BH3 mimetic known to inhibit BCL-2 and BCL-XL leading to the inhibition of proliferation and induction of apoptosis in MM^11,12^.

The histone deacetylase inhibitor (HDACi), Quizinostat specifically targets HDAC6 which regulates cell proliferation and survival^13^. Treatment with Quizinostat reduces the abundance of Protein Phosphatase 3 Catalytic Subunit Alpha (PPP3CA), leading to a reduction in Heat Shock Protein 90  (HSP90), a chaperone protein for HDAC6^14^.

PF-04691502 is an investigational drug that is a member of the phosphatidylinositol 3-kinase (PI3K)/mammalian target of the rapamycin (mTOR) (P13K/mTOR) inhibitor class that has shown anti-proliferative activity in vitro and in vivo^15,16^. The P13K/mTOR signalling pathway has been implicated in cancer cell proliferation motility, growth and survival^17^.

We present an in-depth proteomic profile analysis of 35 CD138^+^ MM samples, both at diagnosis and at relapse. No longitudinal samples were analysed, with the exception of one patient where a diagnostic and a corresponding relapse sample was available. We demonstrate that patients in four different Drug Sensitivity Score (DSS) groups can be alternatively categorised as least/most sensitive to a panel of five drugs. We then performed LC–MS/MS analysis for establishing a proteomic-based biomarker panel to assess sensitivity of these 35 MM patients to give a unique insight into drug resistance to these particular treatments from patients at varying stages of disease progression. In depth analysis of the biological processes associated with the compiled protein lists for each treatment and the top ten most abundant individual proteins for most and least sensitive patients was performed. Such analysis identified a distinct proteomic and chemo-sensitive profile for each patient. The depth and quality of the proteome profiling presented here will enable the discovery of a best possible accurate phenotype of the resistant sub-clones, thus yielding a theranostic profile that will inform therapeutic and drug development strategies.

## Results

### MM patients are stratified into different chemoresistance groups

To determine and examine drug responses of the CD138^+^ plasma cell samples from 35 patients, drug sensitivity scoring (DSS) was used as outlined previously by^18,19^ (see “Drug sensitivity scoring” for more details). Four distinct chemoresistance groups were formed (Supplementary Fig. 1), ranging from sensitive (Group 1) to resistant (Group 4) to the panel of drugs used (Supplementary Fig. 1a), the cytogenetics of which has previously been carried out by^19^ (Supplementary Fig. 2). Twelve patients fell in Group 1, nine in Group 2, eight in Group 3 and six in Group 4. Correlating the DSS with the available clinical data we found that although Group 1 is the most sensitive to treatment, the overall survival (OS) of this group is the shortest (Supplementary Fig. 1b). In contrast, Group 4, although resistant to treatment, exhibits an OS which is similar to that of Group 3 (diminished response to most drugs) and is slightly decreased in comparison to the OS of Group 2 (moderate sensitivities) (Supplementary Fig. 1b). We have determined a more refined approach than stratifying patients as Group 1– Group 4 to a panel of 308 drugs and have further stratified patients as most and least sensitive to each individual therapeutic.

### MM patients show differential response to five different classes of drugs

**Figure 1:**
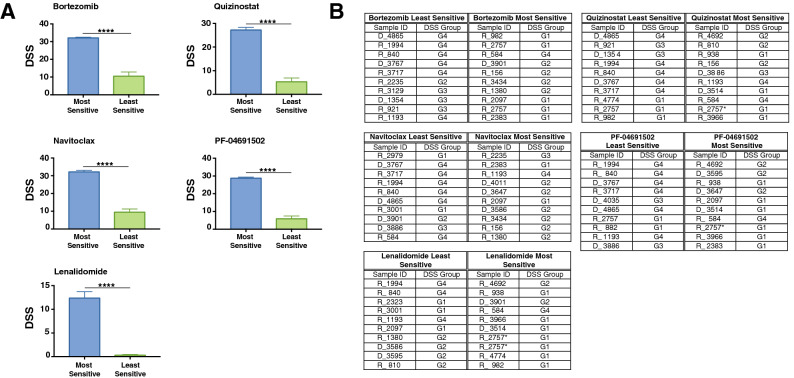
Patients show differential response to five different classes of drugs. (**a**) Most and Least Sensitive patients have a significantly different DSS (p < 0.001) across six drug treatments. (**b**) DSS group of most sensitive and least sensitive patients to six drugs. The least sensitive group was comprised of Group 4 patients whereas the most sensitive group was comprised of patients ranging from Group 1 to Group 3. *The 2nd sample from the patient with longitudinal sampling.

We next investigated the response of the CD138^+^ plasma cell samples from 35 patients, to five anti-myeloma therapies: Bortezomib (PI), Lenolidomide (IMiD), Navitoclax (BCL-2 inhibitor), Quizinostat (HDACi) and the investigational drug PF-04691502. The panel of five drugs were chosen based on their different modes of action, comprising of both well established and investigational MM drugs. Patients were further stratified into groups of “most sensitive” and “least sensitive” to the five different chemotherapeutics used. The most sensitive group is comprised of the ten patients with the highest DSS for each drug and the least sensitive group is comprised of the ten patients with the lowest DSS for each drug (Fig. 1). The comparison between the most and least sensitive patients to individual drugs is significant across all treatments (Fig. 1a). Interestingly, when compiling groups of most sensitive and least sensitive patients to the selected five drugs, the least sensitive group was compiled of Group 4 patients, whereas the most sensitive group was compiled of patients ranging from Group 1 to Group 3 (Fig. 1b).

### Proteomic analysis of patients most/least sensitive to Bortezomib, Quizinostat and PF-04691502 exhibit similar protein signatures

**Figure 2:**
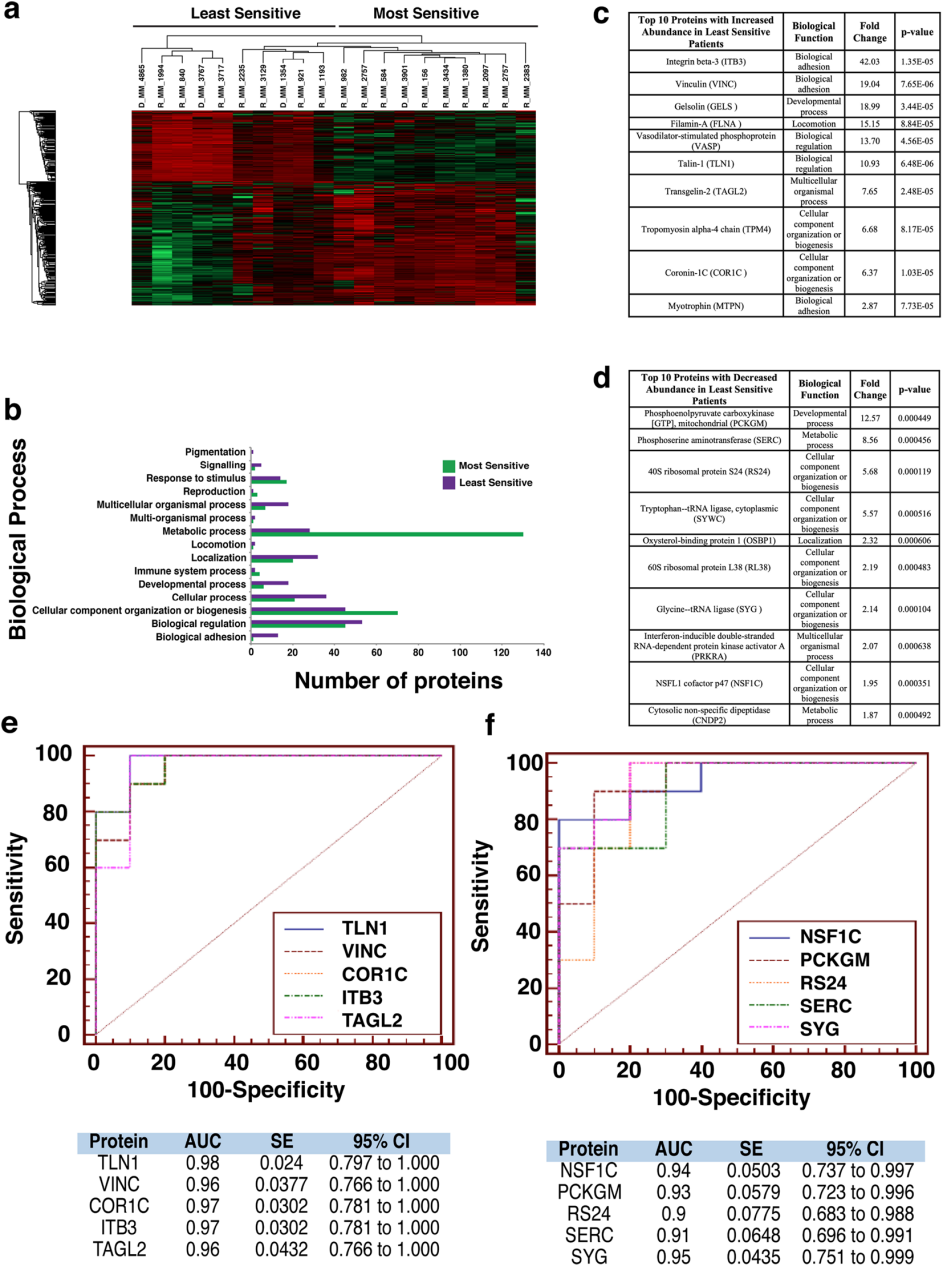
Proteomic analysis of patients following Bortezomib treatment. (**a**) Heatmap showing protein abundance changes of patients most and least sensitive to Bortezomib. Red indicates an increase in protein abundance, while green a decrease in protein abundance. (**b**) Graph showing the biological processes associated with the most and least sensitive patients. (**c**) List of the top ten proteins with increased abundance in the least sensitive patients. The associated biological function, fold change and p-value are also shown. Proteins were designated by p-value and arranged hierarchically by fold change. (**d**) List of the top ten proteins with decreased abundance in the least sensitive patients. The associated biological function, fold change and p-value are also shown. Proteins were designated by p-value and arranged hierarchically by fold change. (**e**) ROC analysis for the top five statistically significant proteins with an increased abundance for the least sensitive patients to bortezomib, including the calculated AUC, standard error (SE) and 95% confidence interval (CI). (**f**) ROC analysis for the top five statistically significant proteins with a decreased abundance for the least sensitive patients to bortezomib, including the calculated AUC, standard error (SE) and 95% confidence interval (CI).

**Figure 3:**
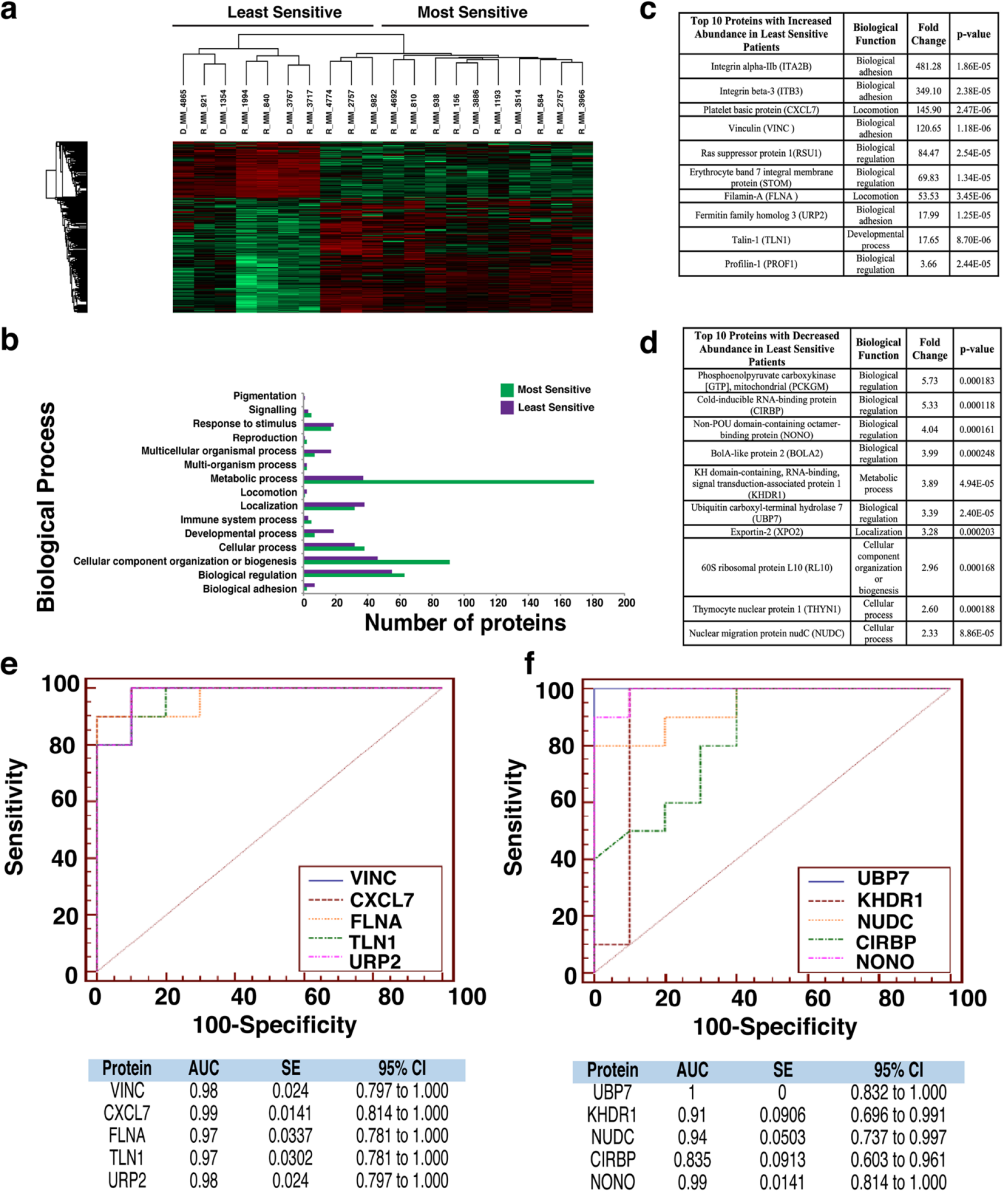
Proteomic analysis of patients following Quizinostat treatment. (**a**) Heatmap showing protein abundance changes of patients most and least sensitive to Quizinostat. Red indicates an increase in protein abundance, while green a decrease in protein abundance. (**b**) Graph showing the biological processes associated with the most and least sensitive patients. (**c**) List of the top ten proteins with increased abundance in the least sensitive patients. The associated biological function, fold change and p-value are also shown. Proteins were designated by p-value and arranged hierarchically by fold change. (**d**) List of the top ten proteins with decreased abundance in the least sensitive patients. The associated biological function, fold change and p-value are also shown. Proteins were designated by p-value and arranged hierarchically by fold change. (**e**) ROC analysis for the top five statistically significant proteins with an increased abundance for the least sensitive patients to Quizinostat, including the calculated AUC, standard error (SE) and 95% confidence interval (CI). (**f**) ROC analysis for the top five statistically significant proteins with a decreased abundance for the least sensitive patients to Quizinostat, including the calculated AUC, standard error (SE) and 95% confidence interval (CI).

**Figure 4:**
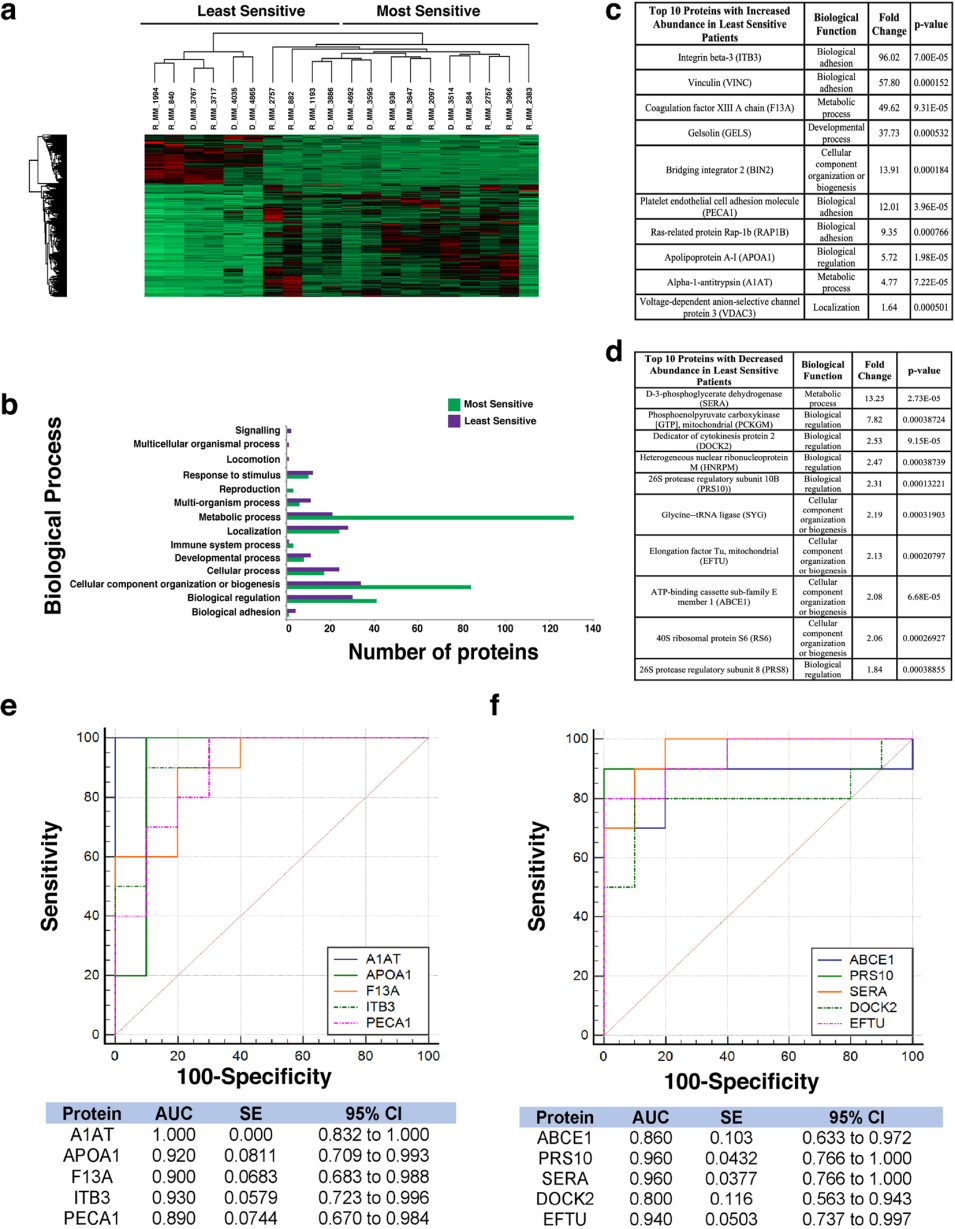
Proteomic analysis of patients following PF-04691502 treatment. (**a**) Heatmap showing protein abundance changes of patients most and least sensitive to PF-04691502. Red indicates an increase in protein abundance, while green a decrease in protein abundance. (**b**) Graph showing the biological processes associated with the most and least sensitive patients. (**c**) List of the top ten proteins with increased abundance in the least sensitive patients. The associated biological function, fold change and p-value are also shown. Proteins were designated by p-value and arranged hierarchically by fold change. (**d**) List of the top ten proteins with decreased abundance in the least sensitive patients. The associated biological function, fold change and p-value are also shown. Proteins were designated by p-value and arranged hierarchically by fold change. (**e**) ROC analysis for top five statistically significant proteins with an increased abundance for the least sensitive patients to PF-04691502, including the calculated AUC, standard error (SE) and 95% confidence interval (CI). (**f**) ROC analysis for the top five statistically significant proteins with a decreased abundance for the least sensitive patients to PF-04691502, including the calculated AUC, standard error (SE) and 95% confidence interval (CI).

In-depth proteomic analysis of samples identified statistically significant (p < 0.05) proteins with changes in abundance. This data was used to compile a heatmap for each individual drug (Figs. 2a, 3a and 4a). Patients exhibited similar protein signatures to Bortezomib (Fig. 2), Quizinostat (Fig. 3) and PF-04691502 (Fig. 4).

Bortezomib (Fig. 2a) shows a clear distinction in protein abundance between the ten most sensitive patients and the ten least sensitive patients. Quizinostat (Fig. 3a) exhibits distinct difference in protein abundance between the two different patient groups, especially in the first seven patients in the least sensitive group in comparison with the most sensitive group. The difference seen in two of the least sensitive patients may be due to the partial positive response seen by the specific three patients in the least sensitive group. With a less apparent distinction between both groups compiled after treatment with PF-04691502 (Fig. 4a), the slight overlap from four of the least sensitive patients into the most sensitive group is most likely due to a partially positive response recorded from these four patient samples, similar to that seen in Quizinostat.

### Proteomic analysis of patients most/least sensitive to Lenalidomide and Navitoclax exhibit different protein signatures

**Figure 5:**
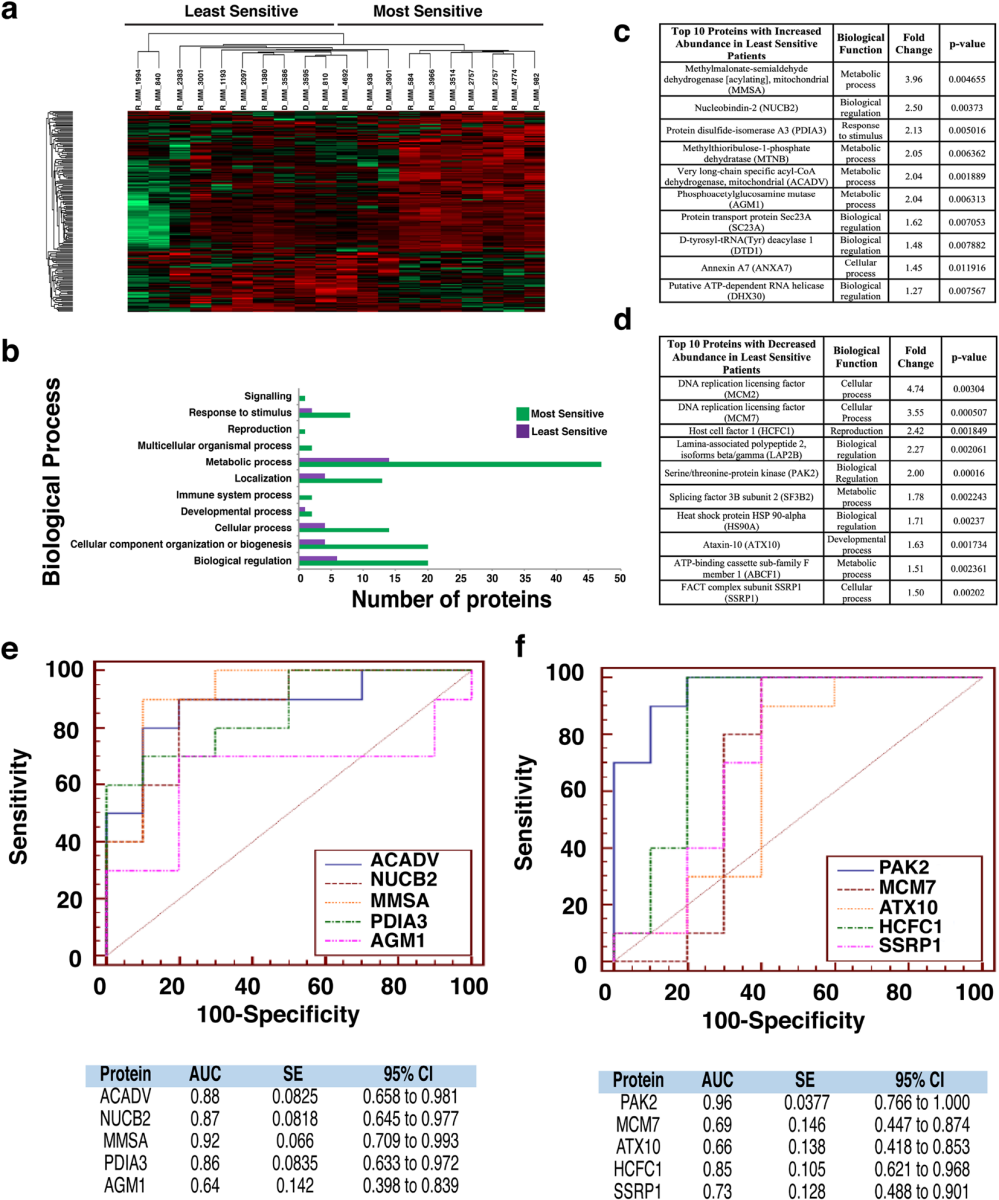
Proteomic analysis of patients following Lenalidomide treatment. (**a**) Heatmap showing protein abundance changes of patients most and least sensitive to lenalidomide. Red indicates an increase in protein abundance, while green a decrease in protein abundance. (**b**) Graph showing the biological processes associated with the most and least sensitive patients. (**c**) List of the top ten proteins with increased abundance in the least sensitive patients. The associated biological function, fold change and p-value are also shown. Proteins were designated by p-value and arranged hierarchically by fold change. (**d**) List of the top ten proteins with decreased abundance in the least sensitive patients. The associated biological function, fold change and p-value are also shown. Proteins were designated by p-value and arranged hierarchically by fold change. (**e**) ROC analysis for the top five statistically significant proteins with an increased abundance for the least sensitive patients to Lenalidomide, including the calculated AUC, standard error (SE) and 95% confidence interval (CI). (**f**) ROC analysis for the top five statistically significant proteins with a decreased abundance for the least sensitive patients to Lenalidomide, including the calculated AUC, standard error (SE) and 95% confidence interval (CI).

**Figure 6:**
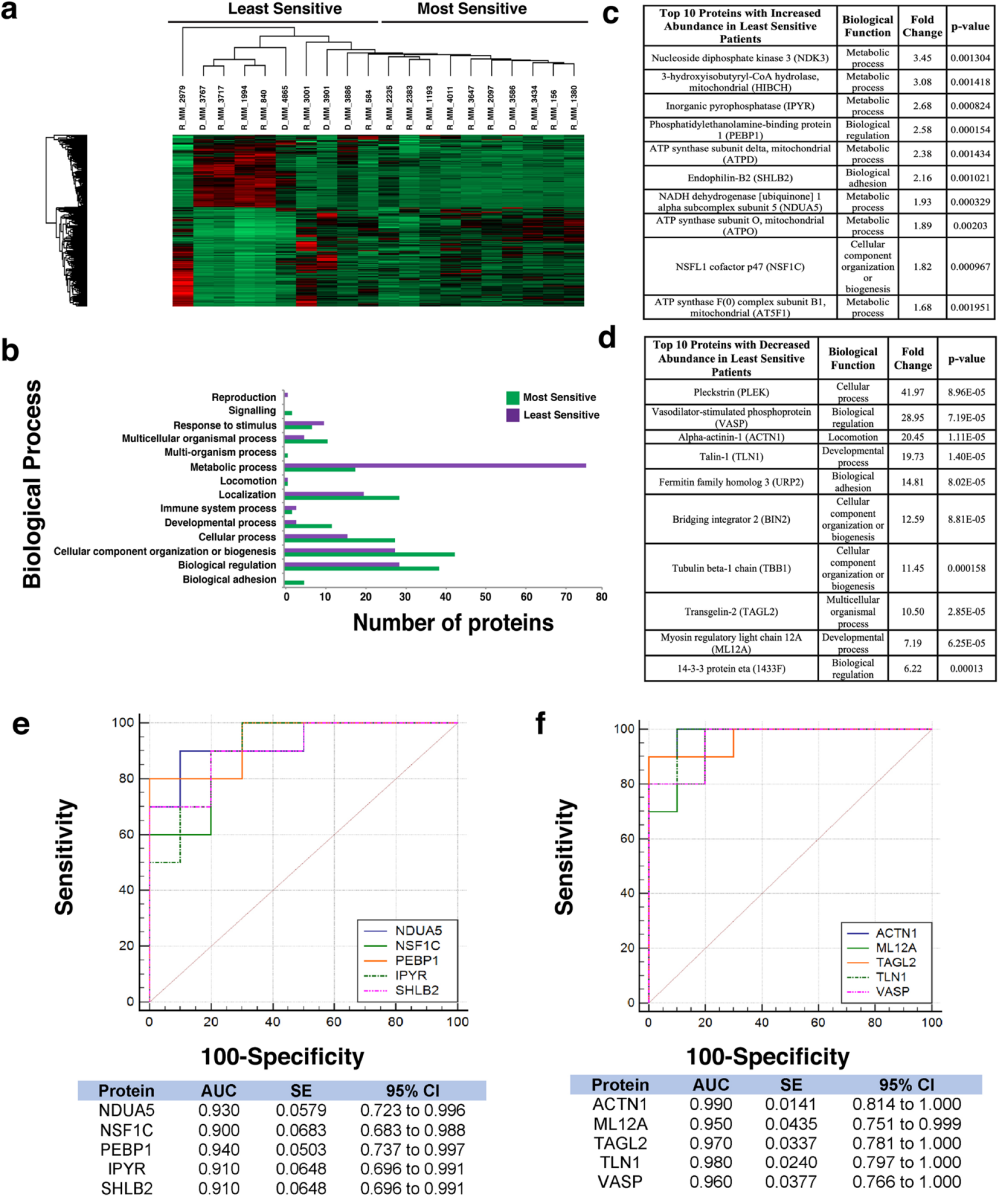
Proteomic analysis of patients following Navitoclax treatment. (**a**) Heatmap showing protein abundance changes of patients most and least sensitive to navitoclax. Red indicates an increase in protein abundance, while green a decrease in protein abundance. (**b**) Graph showing the biological processes associated with the most and least sensitive patients. (**c**) List of the top ten proteins with increased abundance in the least sensitive patients. The associated biological function, fold change and p-value are also shown. Proteins were designated by p-value and arranged hierarchically by fold change. (**d**) List of the top ten proteins with decreased abundance in the least sensitive patients. The associated biological function, fold change and p-value are also shown. Proteins were designated by p-value and arranged hierarchically by fold change. (**e**) ROC analysis for the top five statistically significant proteins with an increased abundance for the least sensitive patients to Navitoclax, including the calculated AUC, standard error (SE) and 95% confidence interval (CI). (**f**) ROC analysis for the top five statistically significant proteins with a decreased abundance for the least sensitive patients to Navitoclax, including the calculated AUC, standard error (SE) and 95% confidence interval (CI).

A distinction between least sensitive and most sensitive patients is less apparent in response to Lenalidomide (Fig. 5a). Navitoclax on the other hand, revealed a stark contrast between most and least sensitive patients’ protein abundance (Fig. 6a).

### Metabolic pathways are associated with most sensitive patients while biological adhesion is associated with least sensitive patients

The proteomic dataset was further analysed using PANTHER to identify the biological processes which are associated with these altered proteins for the five selected chemotherapeutics. For Bortezomib treatment (Fig. 2b) a significant increase in the abundance of proteins related to metabolic processes in the most sensitive group of patients was identified, whereas an increased abundance of proteins associated with biological adhesion was found in the least sensitive group. For Quizinostat treatment (Fig. 3b), an increase in metabolic process-related proteins and cellular component organization or biogenesis proteins is recorded in the most sensitive group. Similar results were obtained for PF-04691502 (Fig. 4b). Biological adhesion associated proteins are increased in abundance following Quizinostat and PF-04691502 treatments in the least sensitive patients.

Metabolic process-related proteins exhibit a higher abundance in the most sensitive patients after treatment using Quizinostat (Fig. 3b), mirroring the findings observed for Bortezomib and PF-04691502. Interestingly, an increased abundance in cellular component organization or biogenesis associated proteins was found in the most sensitive patients following Quizinostat treatment, showing a similar increase as for Navitoclax (Fig. 6b). Again, biological adhesion associated proteins are clearly associated with the least sensitive patients foblowing Quizinostat treatment. A significant increase in metabolic process, cellular process, biological regulation proteins and cellular component organization or biogenesis proteins was observed for Lenalidomide in the most sensitive patients (Fig. 5b); however the larger volume of proteins exhibited in the most sensitive patients may lead to this increased abundance. Furthermore, a significant increase in the abundance of metabolic process proteins was observed in the least sensitive patients foblowing Navitoclax treatment (Fig. 6b).

### Similar individual protein signatures are exhibited for patients treated with Bortezomib, Quizinostat and PF-04691502

We then investigated the individual proteins that showed an increased and decreased abundance in the least sensitive group across the panel of five drugs. Altered proteins associated with Bortezomib treatment led to the observation of fold changes as high as 12.57 for Phosphoenolpyruvate carboxykinase [GTP]. Glycine-tRNA ligase and 40S ribosomal protein S24 were observed to have high statistical significance (p=0.000104). Larger fold changes were recorded with increased abundance in the least sensitive group, as observed for Integrin β3 (42.03-fold). High statistical significance was also recorded with increased abundance in the least sensitive group, such as Talin-1 (p=6.48E^-6^) (Fig. 2c). Five of the ten most statistically significant proteins with decreased abundance in the least sensitive patients were strongly associated with cellular component organization or biogenesis, specifically Glycine-tRNA Ligase, 40S Ribosomal Protein S24, NSFL1 Cofactor p47, 60S Ribosomal Protein L38 and Tryptophan-tRNA Ligase (cytoplasmic) (Fig. 2d).

From the two lists compiled after Quizinostat treatment, a fold increase as high as 5.73 for Phosphoenolpyruvate Carboxykinase was recorded. Ubiquitin carboxyl-terminal hydrolase 7 was recorded as decreased in abundance in the least sensitive group of patients with high statistical significance (p=2.4E^-5^) (Fig. 3d). An increased abundance with a fold change as high as 481.28 was recorded for Integrin alpha-IIb. Vinculin was recorded as increased in abundance in the least sensitive group with high statistical significance (p=1.18E^-6^) (Fig. 3c). Remarkably, five of the ten proteins with increased abundance in the least sensitive group were also recorded as being highly abundant when investigating Bortezomib (Fig. 2c). In addition, two of the ten proteins were also recorded after treatment with PF-04691502 (Fig. 4c).

A list of the ten most significant proteins with altered abundance for the least sensitive group was compiled for treatment using PF-04691502. A fold change as high as 13.25 for d-3-phosphoglycerate dehydrogenase was observed, along with high statistical significance (p=2.73E^-5^), with a decreased abundance in the least sensitive group of patients (Fig. 4d). An increase in abundance with a fold change as high as 96.02 for Integrin-β3 was recorded. Apolipoprotein A-I was observed to have high statistical significance (p=1.98E^-5^) with an increased abundance in the least sensitive group (Fig. 4c). Five of the ten proteins observed with an increase in abundance were also recorded to have an increase in abundance in the least sensitive patients for Bortezomib (Fig. 2c) and Quizinostat (Fig. 3c).

In three of the five drugs tested, there is a very clear increase in the abundance of proteins related to the focal adhesion pathway, specifically actin production leading to cell motility, in the least sensitive groups. Bortezomib (Fig. 2c), Quizinostat (Fig. 3c) and PF-04691502 (Fig. 4c) all showed this statistically significant increased p-values for the abundance of these associated proteins. This indicated that there is a significant change in the production of actin and, consequently, cell mobility related to poor sensitivity to these varying drug treatments. Vinculin and Integrin β3 have a significant increase in abundance in all three of the previously mentioned drugs. A very significant fold increase was recorded in the abundance of Vinculin with Quizinostat treatment, with the lowest of the fold increase abundances seen in treatment with Bortezomib. Integrin β3 has a similar fold increase abundance across all three treatments, the highest of which was observed using Quizinostat treatment and the lowest with Bortezomib treatment.

Talin-1, Gelsolin, Filamin A were all increased in abundance in the least sensitive patients in three of the five drugs tested, specifically in Bortezomib (Fig. 2c), Quizinostat (Fig. 3c) and PF-04691502 (Fig. 4c). Talin-1 is seen to have an increased abundance in Bortezomib and Quizinostat treatments, with highest significance recorded for Quizinostat. Interestingly, a decreased abundance of Talin-1 is noted in treatment with Navitoclax, which is in contrast to the findings for the other drugs tested. Gelsolin is observed to have an increased abundance in treatment with Bortezomib and PF-04691502, with the highest significance recorded for PF-04691502. Filamin A shows a similar trend in increased abundance to that of previously discussed proteins, with an increased abundance observed for Bortezomib and Quizinostat.

### Different individual protein signatures are exhibited for patients treated with Lenalidomide and Navitoclax

The lack of distinction observed in the heat map following Lenalidomide treatment (Fig. 5a) is also apparent in the ten most statistically significant proteins of increased (Fig. 5c) and decreased abundance (Fig. 5d) in the least sensitive patients, where the fold changes and p-values of the abundantly changed proteins in both groups is significantly less drastic to that of the fold changes and p-values recorded for Bortezomib (Fig. 2c,d). Both fold changes and p-values are vastly different to those generated for different drugs within this study, with high statistical significance for Serine/threonine-protein kinase PAK 2 (p=0.00016) and fold changes as high as 4.74 for DNA replication licensing factor MCM2 with decreased abundance in the least sensitive group. High statistical significance for Very long-chain specific acylCoA dehydrogenase, mitochondrial (p=0.001889) and fold changes as high as 3.96 for Methylmalonate-semialdehyde dehydrogenase [acylating], mitochondrial with increased abundance in the least sensitive group recorded. The proteins with altered abundance associated with this particular drug show no obvious overlap with the altered proteins from previously discussed treatments.

Fold increases as high as 41.97 for Pleckstrin and high statistical significance for Alpha-actinin-1 (p=1.11E^-5^) were recorded with decreased abundance in the least sensitive group for Navitoclax (Fig. 6d). Fold changes as high as 3.45 for Nucleoside diphosphate kinase 3 and high statistical significance for Phosphatidylethanolaminebinding protein 1 (p=0.000154) were recorded with increased abundance in the least sensitive group (Fig. 6c), following Navitoclax treatment. Seven out of ten of the most significant proteins increased in abundance in the least sensitive patients are observed to be metabolic process-associated proteins, whereas decreased proteins in the least sensitive group have a less defined involvement in biological processes. Interestingly, trends exhibited after treatment with Navitoclax with an altered protein abundance are opposite to those shown after treatment with Bortezomib, Quizinostat and PF-04691502, with proteins decreased in abundance in the least sensitive patients (Fig. 6d).

### AUC values exhibited by least sensitive patients for Bortezomib, Quizinostat and PF-04691502 

The area under the receiver‐operator characteristic curve (AUC ROC) value for the top five most significant candidate biomarkers was calculated for each drug used in this study. The AUC was found to have good discriminatory power for all biomarkers in the Bortezomib treatment, ranging from 0.9 for RS24 to 0.95 for SYG (Fig. 2f) in proteins with decreased abundance, according to guidelines published by Hosmer & Lemeshow^20^. For the increased abundance proteins in the least sensitive patients, AUC values ranged from 0.96 for VINC and TAGL2 to 0.98 for TLN1, exhibiting remarkable discriminatory power for these potential biomarkers (Fig. 2e). Quizinostat was found to have a range of AUC values of 0.835 for CIRBP to 1.000 for UBP7 for the least sensitive group of patients with decreased abundance (Fig. 3f), and a range from 0.970 for FLNA and TLN1 to 0.99 for CXCL7 in the least sensitive patients with an increased abundance (Fig. 3e). PF-04691502 showed similar notable AUC values ranging from 0.80 for DOCK2 to 0.96 for two of the remaining four potential biomarkers with a decreased abundance in the least sensitive patients (Fig. 4f), and a range of 0.89 for PECA1 to 1.0 for A1AT (Fig. 4e) with respect to increased abundance in the least sensitive patients. These values represent excellent discriminatory power.

### AUC values exhibited by least sensitive patients for Lenalidomide and Navitoclax 

**Figure 7:**
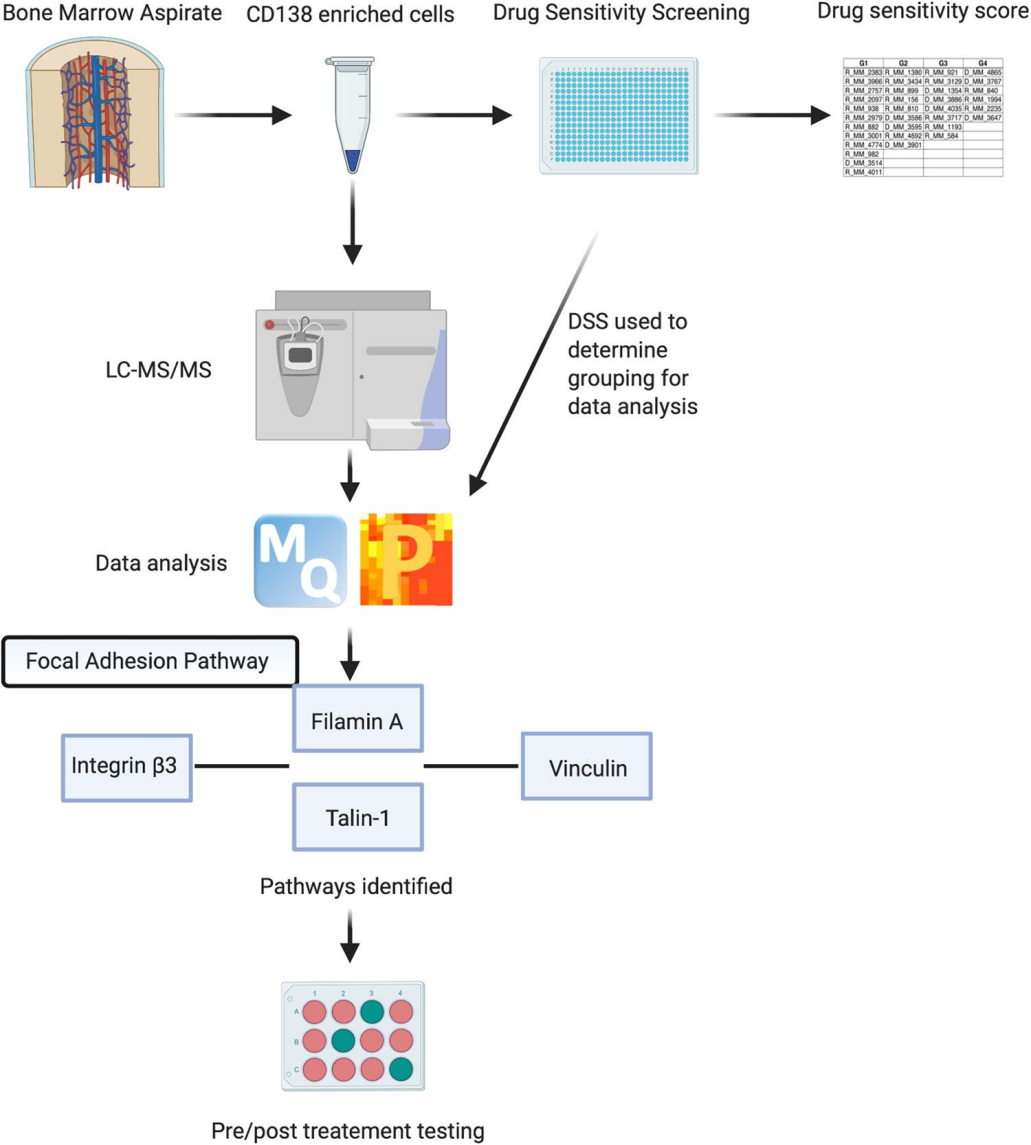
Summary. Figure depicts a workflow of the methods used throughout this study. The relationship between Integrin β3, Filamin A, Talin-1 and Vinculin in the focal adhesion pathway is depicted within the figure. The desired pre/post treatment assay is depicted by the 12-well plate at the end of the work flow, where green depicts most sensitive and pink depicts least sensitive samples.

A broader range of AUC values are observed after treatment using Lenalidomide, with values ranging from 0.66 for ATX10 to 0.96 for PAK2 with regards to decreased abundance in the least sensitive patients (Fig. 5f), and 0.64 for AGM1 to 0.92 for MMSA in the least sensitive patients with an increased abundance (Fig. 5e). Navitoclax reveals a more obvious discriminatory power as a range from 0.95 for ML12A and 0.99 for ACTN1 with decreased abundance in the least sensitive patients (Fig. 6f) and 0.9 for NSF1C and 0.94 for PEBP1 in the least sensitive patients with an increase in abundance (Fig. 6e). All methods and results have been summarised graphically (Fig. 7).

## Discussion

In this paper, the changes in protein abundance for 35 patients to five MM drug treatments was studied to give a unique insight into drug resistance to these particular treatments from patients at varying stages of disease progression, with the long-term goal of developing individual treatment courses catering to individual patient needs (Supplementary Tables 1 and 2). A panel of five drugs was investigated, including well established treatments and investigational treatments. Investigational drugs were included in the study to potentially identify therapeutics for the treatment of MM, which have already been established in other diseases. Three (Bortezomib, Quizinostat and PF-04691502) of the five drugs selected exhibited a similar protein signature while the remaining two drugs (Lenalidomide and Navitoclax) exhibited differing signatures. Bortezomib, Quizinostat and PF-04691502 led to an increased abundance of Vinculin and Integrin β3, while Bortezomib, and Quizinostat showed an increased abundance of Talin-1, Gelsolin and Filamin A, in the least sensitive patients.

Integrin β3 is one of two most notable integrins involved in tumour proliferation and has been implicated in multiple types of cancer including ovarian cancer ^21^. Cell adhesion mediated drug resistance (CAM-DR) has been strongly linked with the increased abundance of Integrin β3. Talin-1, a central component of integrin adhesion and a prerequisite for assembly and maintenance of integrin based cell-extracellular matrix binding^22^, has been seen to exhibit binding sites for Actin and Vinculin^23^. The close association between Talin-1 and the other focal adhesion proteins mentioned in the least sensitive patients to Bortezomib, Quizinostat and PF-04691502, further confirms the role of focal adhesions, actin production and, subsequently, cell motility, previously implicated in cell adhesion in MM cells. It has been recorded that Talin-1-silenced MM cells are notably more susceptible to Bortezomib mediated cell apoptosis^24^. Vinculin has previously been observed to require Talin-1 as a binding partner to comprehensively unmask binding sites for the continuation of Vinculin localisation to focal adhesions^25^. Similar to Talin-1, Gelsolin and Filamin A are associated with actin assembly and actin binding respectively.

The identification of proteins related to the focal adhesion pathway further validated the use of OMICs based techniques to identify potential targets and biomarkers of drug resistance in MM, along with solidifying current techniques used in this particular study. The focal adhesion pathway, along with CAM-DR has commonly been linked to drug resistance in MM patients^26^, due partly to the increased abundance of Wnt3. This increased abundance is directly correlated to the increased abundance of Vinculin and subsequent rearrangement of the actin filaments in MM cells^27^. Vinculin has also been implicated in the increased cell “stiffness” in chemoresistant cells via mechanical cytoskeletal alterations^28^. The increased activation of NFκB, a transcription factor regularly seen to play a role in tumour progression, growth and chemoresistance^29^, and increased levels of Integrin β3 have been reported in multiple studies as causes for drug resistance in cancer cells. Interestingly, it has been observed in multiple studies that targeting NFκB signalling pathways when treating MM, reduces drug resistance to PIs^30^, suggesting that the significantly increased abundance of Integrin β3 shown in this study may be leading to increased levels of NFκB signalling, causing CAM-DR to three of the five drugs. Furthermore, after treatment using Bortezomib, MM cell adhesion to bone marrow stromal cells has been noted to lead to IL-6 secretion, subsequently leading to the activation of NFκB within these stromal cells^31^. This leads to the activation of pathways related to proliferation and cell survival in MM cells^3,26^. This, in turn, leads to the hypothesis that NFκB expression is upregulated following Bortezomib, Quizinostat and PF-04691502 treatment. Due to the discriminatory power identified after AUC ROC curve analysis of the potential biomarkers in this study, the specificity and sensitivity of these potential markers is predicted to be of great use for treatment of MM clinically, for predicting patient response to treatment and patient monitoring, and for the development of future therapeutics. The identification of proteins previously implicated in drug resistance in MM provides proof of concept that OMICs based techniques, coupled with drug sensitivity and resistance testing, gives further insight into disease mechanisms and has the ability to aid in response prediction of patients to treatment combinations. The concept of predicting patient response prior to treatment further drives the possibility of establishing personalised treatment for the needs of each individual.

The stratification of patients included in the study into four distinct chemoresistance groups, ranging from sensitive (Group 1) to resistant (Group 4) to the panel of drugs used, was established. Interestingly, although Group 1 were regarded as sensitive to treatment it was observed that the average OS was lowest for Group 1, in comparison to Group 4 (treatment resistant). It is hypothesised that this decreased OS is due to, firstly, an increase in genomic aberrations observed in Group 1 patients (Supplementary Fig. 2) and, secondly, due to an increase in the abundance of proliferation related proteins observed in Group 1 in comparison to Group 4^19^. This, therefore, led to further stratification of patients into “most sensitive” and “least sensitive” groups, compiled in relation to response to each individual treatment (Fig. 1). The varying response from each patient to each individual treatments further solidifies the need for personalised treatment regimes as a mixed response was recorded for each drug with regards to initial stratification (Fig. 1b). Interestingly, it has been noted recently that increased expression of NFκBIZ (nuclear factor of kappa light polypeptide gene enhancer in B-cell inhibitor zeta), induced by treatment using HDACi, has been linked to an overall good prognosis^32^. As patients in the least sensitive group to Quizinostat treatment are comprised predominantly of Group 4 patients, although they are considered “resistant” to treatment, an increased OS is associated with this group, therefore leading to a better prognosis.

**Figure 8:**
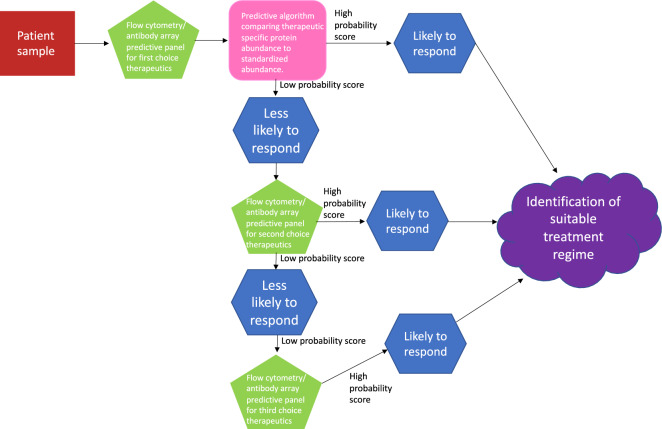
Predictive panel flowchart. Figure depicts the path which clinical samples would take in determining the best choice of personalised treatment per patient, outlined in our manuscript. Decisions would be based on a predictive algorithm, where abundance levels for therapeutic specific proteins would be compared to standardised protein abundances, and a probability score provided. High probability score would indicate the patient would likely respond to the selected therapy, low probably would indicate the patient is less likely to respond to the selected therapy. This information is then used as part of the decision-making process around therapeutic choice. When a patient receives a low probability score for a specific therapy, other predictive panels could be investigated, to try and identify a specific therapy that would give a high probability score, helping with decision making.

The combination of genomic analysis with state-of-the-art proteomics has previously been comprehensively reviewed by Guang et al., indicating that overcoming drug resistance in MM leading to the establishment of personalised treatment, lies here^6^. Proteomic based analysis, coupled with drug sensitivity and resistance screening can provide a theranostic approach to patient treatment, as evident in our findings. Furthermore, this combination has the potential to guide treatment decisions providing personalised regimes and a means to monitor response as well as a deeper understanding of the disease and drug mechanisms. Future work will include the establishment of antibody^33^/mass spectrometry-based^34^ screening assays to identify the different resistant phenotypes at diagnosis/relapse to individual standard of care therapeutics. This study indicates that a panel of biomarkers has the potential to be used clinically, both pre and post treatment, to monitor patient response and predict the best course of treatment (Fig. 8). This could be possible using an ELISA-type assay to a panel of specific biomarkers, indicating early resistance or response to treatment, based on both patient response and proteomic signatures^35^.

## Methods

### Patients and samples

The ethics committee of the Mater Misericordiae University Hospital approved the study in compliance with the Declaration of Helsinki. Informed consent was obtained from all participants involved in the study. A total of 35 patient bone marrow (BM) aspirates were collected from 10 diagnostic and 25 relapse patients. No longitudinal samples were analysed, with the exception of patient 2757. Patient characteristics and associated treatments are detailed in Supplementary Tables 1 and 2. Patients cytogenetics are shown in Supplementary Fig. 2. No exclusion criteria were applied to the patients and the samples were collected prospectively. Data collection was continued at successive relapses to follow disease progression.

### Drug sensitivity scoring

CD138^+^ plasma cells were enriched using the EasySep Human CD138 Positive Selection kit (StemCell Technologies, Grenoble, France) from the mononuclear cell fraction of BM aspirates following gradient separation (Ficoll-Paque PREMIUM; GE Healthcare, Little Chalfont, Buckinghamshire, UK). Drug sensitivity scoring (DSS) was performed based on methods described previously^18,19^. CD138^+^ plasma cells derived from myeloma patients were tested against 308 compounds at five concentrations over tenfold dilutions covering a 10,000-fold concentration range (1–10,000 nM). The drug panel included approved oncology drugs (n = 141) and investigational compounds (n = 167) targeting multiple signalling networks and molecular targets. In brief, 5 μl of cell culture medium comprised of RPMI 1640 medium supplemented with 10% foetal bovine serum, 2 mM L-glutamine, penicillin (100 U/ml), streptomycin (100 μg/ml) and 25% conditioned medium from the HS-5 human BM stromal cell line was added to 384 well drug plates and shaken for 5 min to dissolve the compounds. CD138^+^ plasma cells were diluted in the culture medium and 20 μl of the cell suspension containing 5000 cells was transferred to each well using a MultiDrop Combi peristaltic dispenser (Thermo Scientific, Waltham, MA, USA). The plates were incubated in a humidified environment at 37 °C and 5% CO2. Cell viability was measured after 72 h using the CellTiter-Glo assay (Promega, Madison, WI, USA) with a PHERAstar microplate reader (BMG-Labtech, Offenburg, Germany) to measure luminescence. The mean viability of untreated cells at day three was 124 ± 10.40%. The data was normalized to negative (DMSO only) and positive control wells (containing 100 μM benzethonium chloride).

### Protein digestion

CD138^+^ enriched plasma cells were initially lysed in RIPA buffer (25 mM Tris, pH 7–8; 150 mM NaCl; 0.1% SDS; 0.5% sodium deoxycholate and 1% NP-40). The lysates were buffer exchanged using the 'filter aided sample preparation' (FASP) method in a buffer containing 8 M urea/50 mM NH4HCO3/0.1% ProteaseMax. The protein amount was estimated using an RC/DC protein assay from Bio-Rad. BSA was used as a standard. After dithiothreitol reduction and iodoacetic acid-mediated alkylation, a double digestion was performed using Lys-C (for 4 h at 37 °C) and Trypsin (overnight at 37 °C) on 5 µg of protein. Digested samples were desalted prior to analysis using C18 spin columns (Thermo Scientific, UK)^36^.

### Mass spectrometry

500 ng of each digested sample was loaded onto a Q-Exactive (ThermoFisher Scientific, Hemel Hempstead, UK) high-resolution accurate mass spectrometer connected to a Dionex Ultimate 3000 (RSLCnano) chromatography system (ThermoFisher Scientific, Hemel Hempstead, UK). Peptides were separated using a 2% to 40% gradient of acetonitrile on a Biobasic C18 Picofrit column (ThermoFisher Scientific, Hemel Hempstead, UK) (100 mm length, 75 mm ID) over 65 min at a flow rate of 250 nl/min. Data was acquired with the mass spectrometer operating in automatic data dependent switching mode. A full MS scan at 140,000 resolution and a range of 300–1700 m/z was followed by an MS/MS scan, resolution 17,500 and a range of 200–2000 m/z, selecting the ten most intense ions prior to MS/MS^37^.

### Data analysis

Protein identification and label-free quantification (LFQ) normalisation of MS/MS data was performed using MaxQuant v1.5.2.8 (http://www.maxquant.org). The Andromeda search algorithm incorporated in the MaxQuant software was used to correlate MS/MS data against the *Homo sapiens* Uniprot reference proteome database and a contaminant sequence set provided by MaxQuant. Perseus v.1.5.6.0 (www.maxquant.org/) was used for data analysis, processing and visualisation. Normalised LFQ intensity values were used as the quantitative measurement of protein abundance for subsequent analysis. The data matrix was first filtered for the removal of contaminants and peptides identified by site. LFQ intensity values were log2 transformed and each sample was assigned to its corresponding group. ANOVA-based multisample t-test was performed using a cut-off of p < 0.05 on the post imputated dataset to identify statistically significant differentially abundant proteins^38^. Receiver‐operating characteristic (ROC) curve analysis was performed as it is a useful tool for the assessment of biomarker accuracy. The ROC plots were obtained by plotting all sensitivity values (true positive fraction) on the y‐axis against their equivalent (100‐specificity) values (false positive fraction) for all available thresholds on the x‐axis (MedCalc for Windows 8.1.1.0, Medcalc Software, Mariakerke, Belgium). The area under the curve (AUC) was calculated to provide a summary of overall classifier effectiveness. In our study, we consider AUC values ranging from 0.5 to 0.7 as poor, 0.7–0.8 as average, 0.8–0.9 as good and > 0.9 as outstanding^39^.

### Bioinformatics analysis

In order to group identified proteins based on their protein class and to identify potential protein targets with increased abundance in both most and least sensitive patients, the publicly available bioinformatics software PANTHER was employed (http://pantherdb.org/).

## Supplementary Information

Supplementary Information.
