# Supplementary material for: Next generation proteomics with drug sensitivity screening identifies sub-clones informing therapeutic and drug development strategies for multiple myeloma patients
Source: Sci Rep. 2021 Jun 18;11:12866. doi: 10.1038/s41598-021-90149-y (PMC8213739; doi:10.1038/s41598-021-90149-y)
Supplement: Supplementary file 1 — Supplementary Information. [file 41598_2021_90149_MOESM1_ESM.docx]

**Combining Next Generation Proteomics Platforms with Drug Sensitivity Resistance Testing allows Identification of Physiologically Distinct Sub-clones that can inform Therapeutic and Drug Development Strategies for patients with Multiple Myeloma**

Ciara Tierney^1^, Despina Bazou^2^, Muntasir M. Majumder^3^, Pekka Anttila^4^, Raija Silvennoinen^4^, Caroline A. Heckman^3^, Paul Dowling^1^, Peter O’Gorman^2^

**Corresponding author:**

Prof. Peter O’Gorman

Department of Hematology,

Mater Misericordiae University Hospital,

Dublin, Ireland

Email: [pogorman@mirtireland.com](mailto:pogorman@mirtireland.com)

Tel: 00 353 1 8500977

Fax: 00 353 1 8500962


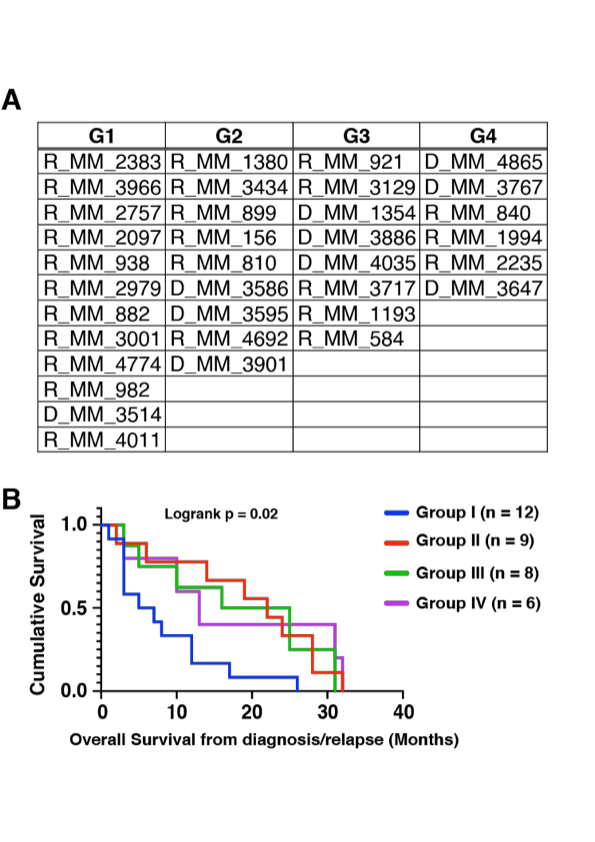
**Supplementary Fig. 1.** Chemoresistance and overall survival of the patient cohort. A) Four distinct chemoresistance groups were formed, ranging from sensitive (G1) to resistant (G4) to the panel of drugs used. B) G1 is the most sensitive to treatment with the shortest OS. G4, although resistant to treatment, exhibit an OS which is similar to that of G3 (diminished response to most drugs) and is slightly decreased in comparison to the OS of G2 (moderate drug sensitivities). D denotes samples were taken at Diagnosis, and R denotes samples taken at Relapse.

**Supplementary Fig. 2.** Cytogenetics of the patient cohort. Heatmap showing the most commonly related cytogenetic abnormalities to MM of the patient cohort.

**Supplementary Table 1.** MM patient cohort characteristics. Table illustrating the gender, age at diagnosis, paraprotein and light chain of the MM patient cohort.

| **Patient ID** | **Gender** | **Age at Diagnosis** | **Paraprotein** | **Light Chain** |
| --- | --- | --- | --- | --- |
| R_MM_2383 | Male | 58 | Unknown | lambda |
| R_MM_3966 | Female | 65 | IgA | lambda |
| R_MM_2757 | Male | 59 | IgA | lambda |
| R_MM_2097 | Female | 59 | Unknown | kappa |
| R_MM_938 | Male | 50 | IgG | kappa |
| R_MM_2979 | Female | 69 | IgA | kappa |
| R_MM_882 | Female | 57 | Unknown | lambda |
| R_MM_3001 | Male | 56 | Unknown | kappa |
| R_MM_4774 | Male | 78 | IgA | kappa |
| R_MM_982 | Male | 56 | IgG | kappa |
| D_MM_3514 | Female | 68 | IgG | kappa |
| R_MM_4011 | Male | 66 | Unknown | Unknown |
| R_MM_1380 | Male | 68 | IgA | lambda |
| R_MM_3434 | Male | 49 | IgG | kappa |
| R_MM_899 | Male | 62 | IgA | lambda |
| R_MM_156 | Female | 62 | IgA | kappa |
| R_MM_810 | Male | 74 | Unknown | Unknown |
| D_MM_3586 | Male | 61 | Unknown | kappa |
| D_MM_3595 | Male | 67 | IgG | lambda |
| R_MM_4692 | Male | 41 | IgG | lambda |
| D_MM_3901 | Male | 71 | IgA | kappa |
| R_MM_921 | Female | 56 | Unknown | lambda |
| R_MM_3129 | Male | 60 | IgG | kappa |
| D_MM_1354 | Male | 66 | Unknown | Unknown |
| D_MM_3886 | Female | 59 | IgG | lambda |
| D_MM_4035 | Female | 61 | IgG | kappa |
| R_MM_3717 | Male | 51 | Unknown | kappa |
| R_MM_1193 | Male | 68 | IgA | lambda |
| R_MM_584 | Male | 71 | IgA | kappa |
| D_MM_4865 | Male | 66 | Unknown | kappa |
| D_MM_3767 | Female | 55 | IgA | lambda |
| R_MM_840 | Female | 64 | IgA | kappa |
| R_MM_1994 | Female | 68 | IgG | lambda |
| R_MM_2235 | Female | 56 | IgG | kappa |
| D_MM_3647 | Male | 63 | IgG | kappa |

**Supplementary Table 2.** MM patient cohort treatment course. Table illustrating the 1^st^ next line treatment, all subsequent line treatments and the deepest response in next line treatments.

| **Patient ID** | **Name of 1st next line treatment** | **Names of all next line treatments** | **Deepest response in next line treatment** |
| --- | --- | --- | --- |
| R_MM_2383 | VAD | VAD | Exitus |
| R_MM_3966 | DR-PACE (Cis/Cpm/Dxm/Dox/Eto/L) | DR-PACE (Cis/Cpm/Dxm/Dox/Eto/Len) | PR |
| R_MM_2757 | Bor/Dxm/Len | Bor/Dxm/Len | PR |
| R_MM_2097 | Len/Dxm | 1. Len/Dxm 2. Bor/Dxm/Len | PR |
| R_MM_938 |  |  |  |
| R_MM_2979 | Bor/Dxm | 1. Bor/Dxm 2. Bor/Mel/Pred (VMP) | VGPR |
| R_MM_882 | Benda/Bor/Pred | Benda/Bor/Pred |  |
| R_MM_3001 | Bor/Dxm/Len | Bor/Dxm/Len | PR |
| R_MM_4774 |  |  |  |
| R_MM_982 | Bor/Dxm | Bor/Dxm | PR |
| D_MM_3514 | Dxm | Dxm | VGPR |
| R_MM_4011 | Radiotherapy |  | VGPR |
| R_MM_1380 | Bor/Dxm/Len | Bor/Dxm/Len | SD |
| R_MM_3434 | Bor/Dxm | Bor/Dxm | PR |
| R_MM_899 | Pomal/Dxm | Pomal/Dxm | SD |
| R_MM_156 | Radiotherapy | 1. Radiotherapy 2. Bor/Dxm/Len 3. Len/Dxm | VGPR |
| R_MM_810 | Bor/Dxm | 1. Bor/Dxm 2. Bor/Dxm/Len | Clinical Relapse |
| D_MM_3586 | Dxm | Dxm | PR |
| D_MM_3595 | Bor/Dxm/Len | 1. Bor/Dxm/Len 2. Mobilisation (Cpm/G-CSF) 3. AutoHSCT (HD Mel) 4. Len | Scr |
| R_MM_4692 | Carfilzomib |  |  |
| D_MM_3901 | Bor/Mel/Pred (VMP) | 1. Bor/Mel/Pred (VMP) 2. Bor/Dxm/ 3. Bor/Cpm/Dxm | PR |
| R_MM_921 | Len/Dxm | 1. Len/Dxm 2. Dxm 3. DLI | PR |
| R_MM_3129 | Pomal/Dxm | Pomal/Dxm | SD |
| D_MM_1354 |  |  |  |
| D_MM_3886 | Bor/Dxm | 1. Bor/Dxm 2. Bor/Cpm/Dxm 3. Bor/Dxm/Len 4. Mobilisation (Cpm) 5. Bor/Dxm/Len 6. AutoHSCT (HD Mel) | VGPR |
| D_MM_4035 | Radiotherapy | 1. Bor/Dxm 2. Bor/Cpm/Dxm 3. Bor/Dxm/Len 4. Mobilisation (Cpm) 5. Bor/Dxm/Len 6. AutoHSCT (HD Mel) | VGPR |
| R_MM_3717 | Bor/Dxm | Bor/Dxm | VGPR |
| R_MM_1193 | Bor/Dxm/Len | Bor/Dxm/Len | VGPR |
| R_MM_584 | No treatment | No treatment | Exitus |
| D_MM_4865 |  |  |  |
| D_MM_3767 | Bor/Dxm/Len | 1. Bor/Dxm/Len 2. Mobilisation (G-CSF) | PR |
| R_MM_840 | Len/Dxm | Len/Dxm | VGPR |
| R_MM_1994 | Bor/Cpm/Dxm/Len | Bor/Cpm/Dxm/Len | PD |
| R_MM_2235 |  |  |  |
| D_MM_3647 | Benda/ Bor/Pred | Benda/Bor/Pred | PR |
